# The involvement of AMPK/GSK3-beta signals in the control of metastasis and proliferation in hepato-carcinoma cells treated with anthocyanins extracted from Korea wild berry Meoru

**DOI:** 10.1186/1472-6882-14-109

**Published:** 2014-03-25

**Authors:** Song Yi Park, Yun-Kyoung Lee, Won Sup Lee, Ock Jin Park, Young-Min Kim

**Affiliations:** 1 Department of Biological Sciences and Biotechnology, Hannam University Daedeok Valley Campus, 461-6 Jeonmin-dong, Daejeon 305-811 Yuseong-gu, South Korea; 2 Department of Cell Biology, State University of New York Downstate Medical Center, 450 Clarkson Avenue, Brooklyn, NY 11203USA; 3 Department of Internal Medicine, Institute of Health Sciences, Gyeongsang National University School of Medicine, Gyeongnam Regional Cancer Center, Gyeongsang National University Hospital, Jinju 660-702, South Korea; 4 Department of Food and Nutrition, Hannam University Daedeok Valley Campus, 461-6 Jeonmin-dong, Daejeon 305-811 Yuseong-gu, South Korea

**Keywords:** AMP-activated protein kinase, Glycogen synthase kinase 3-beta, beta-catenin, Meoru origin anthocyanins, Anti-metastatic potential

## Abstract

**Background:**

Activation of the Wnt pathway is known to promote tumorigenesis and tumor metastasis, and targeting Wnt pathway inhibition has emerged as an attractive approach for controlling tumor invasion and metastasis. The major pathway for inhibiting Wnt is through the degradation of β-catenin by the GSK3-beta/CK1/Axin/APC complex. It was found that Hep3B hepato-carcinoma cells respond to anthocyanins through GSK3-beta-induced suppression of beta-catenin; however, they cannot dephosphorylate GSK3-beta without AMPK activation.

**Methods:**

We tested the effects of anthocyanins on proliferation and apoptosis by MTT and Annexin V-PI staining *in vitro*. Mouse xenograft models of hepato-carcinomas were established by inoculation with Hep3B cells, and mice were injected with 50 mg/kg/ml of anthocyanins. In addition, protein levels of p-GSK3-beta, beta-catenin, p-AMPK, MMP-9, VEGF, and Ang-1 were also analyzed using western blot.

**Results:**

Anthocyanins decrease phospho-GSK3-beta and beta-catenin expression in an *in vivo* tumor xenograft model, increase AMPK activity in this model, and inhibit cell migration and invasion, possibly by inhibiting MMP-2 (*in vitro*) and the panendothelial marker, CD31 (*in vivo*). To elucidate the role of the GSK3-beta/beta-catenin pathway in cancer control, we conditionally inactivated this pathway, using activated AMPK for inhibition. Further, we showed that AMPK siRNA treatment abrogated the ability of anthocyanins to control cell proliferation and metastatic potential, and Compound C, an AMPK inhibitor, could not restore GSK3-beta regulation, as exhibited by anthocyanins in Hep3B cells.

**Conclusion:**

These observations imply that the AMPK-mediated GSK3-beta/beta-catenin circuit plays crucial roles in inhibiting cancer cell proliferation and metastasis in anthocyanin-treated hepato-carcinoma cells of Meoru origin.

## Background

Hepato-carcinoma is one of the most common cancers worldwide, and the incidence of new cases has increased in recent years. It is often highly metastatic and resistant to anticancer treatment strategies. One of the key genetic defects that confer resistance against hepato-carcinoma treatment is the mutation of β-catenin, which is a key component of the Wnt signaling pathway [1]. The Wnt pathway is crucial to cell proliferation, differentiation, and survival, and cells with enhanced activation of the Wnt pathway have strong invasive activity [2]. Invasion of cancer cells into surrounding tissue and the vasculature is an initial step in tumor metastasis [3]. Several studies have suggested that over-activation of β-catenin in the cytosol is related to cancer metastasis [4]. Under normal conditions, β-catenin is phosphorylated at Ser33/37 by active glycogen synthase kinase 3β (GSK3β), triggering subsequent β-catenin proteasomal degradation [5]. The active form of GSK3β is in the dephosphorylated state; however, when it is phosphorylated, GSK3β loses its activity and no longer controls β-catenin [6]. Uncontrolled β-catenin is translocated from the cytosol to nucleus, where it activates target genes involved in cell proliferation and metastasis [7]. Recent studies have shown that several phytochemicals, including anthocyanins, can regulate cell growth by regulating the phosphorylation of GSK3β [8]. Anthocyanins from the fruit of *Vitis coignetiae* Pulliat (known as meoru in Korea) are water-soluble flavonoids found in red and blue colored fruits and vegetables, such as blueberry, cranberry, and red cabbage [9,10]. Anthocyanins have been linked to a number of intracellular functions, including cellular redox status modification, free radical scavenging activity, and chelation of metals [11]. In addition, anthocyanins (delphinidin-3,5-diglucoside: cyanidin-3,5-diglucoside: petunidin-3,5-diglucoside: delphinidin-3-glucoside: malvdin-3,5-diglucoside: peonidin-3,5-diglucoside: cyanidin-3-glucoside: petunidin-3-glucoside: peonidin-3-glucoside: malvidin-3-glucoside = 27:63:8.27:1:2.21:2.21:6.7:1.25:5.72:1.25) isolated from *V. coignetiae* Pulliat fruits show anti-invasive effects and apoptotic effects in human hepato-carcinoma cells [12,13]. They also exhibit cancer-preventive effects that occur through their abilities to interfere with the cell signaling pathway [8]. Previous experiments have shown that anthocyanins induce cell cycle blockage at G1/G0 and G2/M phases and regulate the extracellular regulated kinase (ERK), c-Jun N-terminal kinase (JNK), and p38 mitogen-activated protein kinase (MAPK) pathways in several cancer types [14-16]. In addition, anthocyanins have been shown to inhibit the activation of transcription factors such as nuclear factor-κB (NF-κB) and activator protein-1 (AP1) [17].

In this study, we analyzed downstream signals of AMPK to search for naturally originating novel modulators of the AMPK/GSK3β/β-catenin pathway to control cancer cell proliferation and metastasis. We found that anthocyanins activated GSK3β, thereby decreasing β-catenin, and that AMPK was an upstream regulator of GSK3β/β-catenin pathway. This information holds promise for therapeutic modulation of GSK3β/β-catenin-pathway-dependent invasiveness in cancer cells.

## Methods

### Cell culture and reagents

The Hep3B hepato-carcinoma cell line was purchased from the American Type Culture Collection (Manassas, VA) and was cultured in Dulbecco’s modified Eagle’s medium with 10% fetal bovine serum (Gibco, Grand Island, NY). Insulin-like growth factor (IGF)-1, 3-(4,5-dimethylthiazol-2-yl)-2, 5-diphenyltetrazolium bromide (MTT) and Hoechst 33342 were obtained from Sigma (St Louis, MO). Compound C and 6-bromoindirubin-3′-oxime (BIO) were purchased from Calbiochem (San Diego, CA). Monoclonal antibodies specific for p-AMPK (Thr^172^), AMPKα1, p-GSK3β (Ser^9^), GSK3β, β-catenin, Ang-1, VEGF and MMP-9 were purchased from Cell signaling Technology (Beverly, MA, USA). CD31 antibody was purchased from Abcam (Cambridge, UK), and β-actin antibody was obtained from Sigma (St Louis, MO).

### Isolation of anthocyanins from Meoru

Anthocyanins were conducted by Won Sup Lee’s group at Gyeongsang National University School of Medicine. The plant with voucher specimen number KNKA200506031111 was deposited in the Korea national arboretum. Fruit of Meoru was collected in the middle of September 2007 at Jiri mountain in Korea, freeze-dried and stored in dark glass containers at −20°C until required for analysis. Anthocyanins pigments were extracted by maceration of the fruits (100 g) in methanol containing 0.1% HCl at 5°C for 24 h. The extraction procedure was repeated three times. After concentration under reduced pressure (Rotavapor R-124, Buchi, Switzerland), the extract was diluted with distilled water (100 ml) and partitioned against ethyl acetate (100 ml). The water layer containing the pigments was concentrated to 50 ml. The concentrate was purified according to established procedures by means of ethyl acetate/water partitioning and adsorption chromatography on a bed of Amberlite XAD-7 (Sigma, Yongin, South Korea) [18].

### Cell proliferation measurements

Hep3B cells seeded on 96-well microplates at 4 × 10^3^ cells per well were incubated with the anthocyanins at the indicated concentrations for 48 h. Following incubation with the anthocyanins, the medium was removed, and the cells were then incubated with 100 μl MTT solution (2 mg/ml MTT in phosphate-buffered saline (PBS)) for 4 h. The samples were then solubilized in dimethyl sulfoxide and the purple formazan dye, converted from MTT by viable cells, was quantified by absorbance at 560 nm.

### Apoptosis detection

Apoptosis was measured using an FITC-Annexin V apoptosis detection kit (BD Pharmingen™, San Diego, CA) or Hoechst 33342 chromatin staining dye. For Annexin V/propidium iodide staining after treatment with anthocyanins, cells were harvested by trypsinization, washed with ice-cold PBS and suspended in a binding buffer at a density of 1 × 10^6^ cells/ml. Cells were stained with Annexin V-fluorescein isothiocyanate and propidium iodide and analyzed by flow cytometry (Becton-Dickinson Biosciences, Drive Franklin Lakes, NJ). To examine chromatin condensation, cells were stained with 10 μM Hoechst 33342 for 30 min and fixed with 3.7% formaldehyde for 15 min. Changes in chromatin condensation were observed by fluorescence microscopy (Olympus Optical Co., Tokyo, Japan).

### Wound healing assay

Hep3B cells were grown on 6-well plate to 100% confluent monolayer and then scratched to form a 100 μm wound by using sterile pipette tips. The cells were then cultured in the presence or absence of AIMs (400 μg/ml) in serum-free media for 24 h. The images were recorded at 0 h and 48 h after scratch using an Olympus photomicroscope (Olympus Optical Co., Tokyo, Japan).

### Invasion assay

For the cell invasion assays, Hep3B cells were cultured in serum-free media overnight. Cells (5 × 10^4^ cells) were loaded onto pre-coated Matrigel 24-well invasion chambers (BD Biosciences, San Jose, CA) in the presence or absence of anthocyanins. Then 0.5 ml of medium containing 20% FBS was added to the wells of the plate to serve as a chemoattractant. The Matrigel invasion chambers were incubated for 24 h. Invading cells were fixed with 10% formalin, stained with crystal violet, and analyzed according to manufacturer’s instructions.

### Gelatin zymography

The gelatinolytic activities of matrix metalloproteinase (MMP)-2 in the conditioning culture medium were assayed by electrophoresis on 8% polyacrylamide gels containing 0.1% gelatin at 4°C. Polyacrylamide gels were run at 120 V, washed in 2.5% Triton X-100 for 1 h, and then incubated for 16 h at 37°C in activation buffer (50 mM Tris–HCl, pH 7.5, 10 mM CaCl_2_). After staining with Coomassie blue (10% glacial acetic acid, 30% methanol and 0.5% Coomassie brilliant blue) for 2 h, the gel was washed with a solution of 10% glacial acetic acid and 40% methanol without Coomassie blue for 1 h. White lysis zones indicating gelatin degradation were revealed by staining with Coomassie brilliant blue.

### Western blot

After starvation for 12 h in serum-free medium, cells were seeded into six-well plates and treated with test compounds. Total proteins were extracted using a RIPA lysis buffer (50 mM Tris–HCl (pH 8.0), 1% NP-40, 0.5% sodium deoxycholate, 150 mM NaCl and 1 mM phenylmethylsulfonyl fluoride) and subjected to western blot analysis with specific antibodies. The proteins were then visualized by enhanced chemiluminescence (Intron, Kyunggi, Korea) and detected using an LAS4000 chemiluminescence detection system (Fuji, Tokyo, Japan).

### Transient transfection with small interfering RNA

Specific small interfering RNAs (siRNAs)-targeting AMPKα1 (PRKAA1) and mTOR and non-specific control siRNAs were purchased from Dharmacon (Chicago, IL). For transient transfection, cells were seeded at a density of 5 × 10^4^ cells/ml in antibiotic-free medium, and siRNAs were transfected using the DharmaFECT4 transfection reagent (Dharmacon) according to the manufacturer’s instructions. After incubation for 72 h, the cells were analyzed by MTT assay or western blot.

### Tumor formation

Five-week-old male Balb/c nu/nu mice were obtained from SLC (Tokyo, Japan) and housed in sterile filter-topped cages. Hep3B hepato-carcinoma cells (1 × 10^6^ cells/150 μl) were subcutaneously injected into the left flank of the mice. One week after the injection of Hep3B cells, anthocyanins were dissolved in PBS and administered intraperitoneally (50 mg/kg/day) for 20 days. The control animals were injected with vehicle (PBS) alone. Tumor size was measured using a caliper at 2 day intervals, and the volume was calculated by the modified formula V = 1/2 (length × width^2^). After the 20 day treatment, tumors were removed and frozen in liquid nitrogen for western blot analysis or fixed with formalin for immunohistochemistry. All animal experiments were approved by the Ethics Committee for Animal Experimentation, Hannam University.

### Immunohistochemistry

Tumor specimens from mice were fixed in 10% formaldehyde, embedded in paraffin and sectioned into 5 μm thick slices. Sections were deparaffinized with xylene and dehydrated with 98% ethanol. Serial sections were stained using standard immunoperoxidase techniques with primary antibodies against CD31 (1:100) and p-AMPKα1 (1:50). For epitope retrieval, specimens were microwave treated for 25 min before incubation with primary antibodies. Pre-immune serum was used as a negative control for immunostaining, and positive staining was visualized with diaminobenzidine, followed by a light counter-staining with hematoxylin. All findings were evaluated by a pathologist blinded to the treatment conditions, and samples were evaluated on the basis of stain intensity and percentage of reactive cells. Images of representative results were recorded.

### Statistical analysis

Cell viability and tumor volume data were statistically analyzed using unpaired t-test (SPSS, Chicago, IL). P < 0.05 was considered statistically significant.

## Results

### Anthocyanins inhibit cell growth *in vivo* and *in vitro*

**Figure 1:**
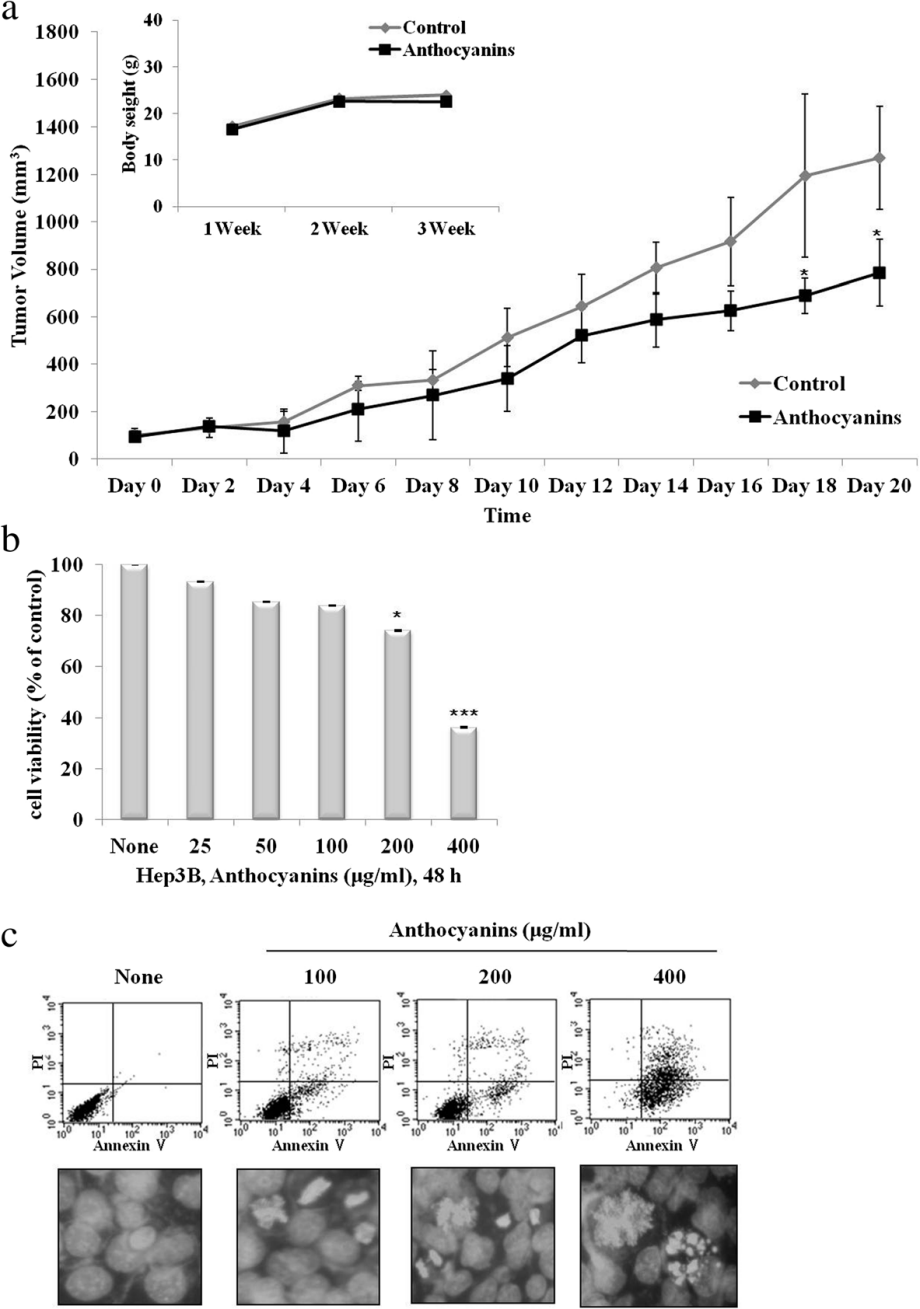
**Anthocyanins suppress tumor growth in a xenograft model and decrease cell growth and apoptosis in Hep3B hepato-carcinoma cells *in vitro*. (a)** Hep3B hepato-carcinoma cells (1 × 10^6^ cells/0.1 ml) were injected subcutaneously into the left flanks of Balb/C nu/nu mice (n = 5 per group). After 1 week, mice received anthocyanins s.c. (50 mg/kg/day) for 20 days. Tumor volume was measured once every 2 days and calculated as described in the Materials and Methods section. Body weight was measured once each week. *P < 0.05, compared with control tumor volume on day 18 or 20. **(b)** Cells were treated with anthocyanins (25–400 μg/ml) for 48 h, and cell viability was measured using the MTT assay. *P < 0.05 and ***P < 0.001, compared with control. **(c)** Anthocyanins-treated cells were stained with Annexin V-FITC, PI or Hoechst 33342 dye (10 μM) and analyzed by flow cytometry or fluorescence microscopy.

To determine whether anthocyanins exert antitumor activity in an *in vivo* model, we examined their effect on tumor growth in a Hep3B xenograft model. Anthocyanins (50 mg · kg^−1^ · day^−1^) was administered intraperitoneally once a day for 20 days, starting 1 week after the initial injection of Hep3B cells. Body weight remained unchanged in all groups, but tumor growth was significantly reduced in the anthocyanin-treated group compared with the control group (Figure 1a). To confirm that anthocyanins could function as chemopreventive agents, we tested their effect on cell viability. We found that anthocyanins inhibited cell growth in a dose-dependent manner (Figure 1b) and induced apoptosis, as measured by Annexin V/propidium iodide (PI) staining and Hoechst 33342 staining (Figure 1c).

### *In vivo* and *in vitro* regulation of AMPK, GSK3β and β-catenin, and cell growth effects through inhibition of AMPK in anthocyanin-treated Hep3B cells

**Figure 2:**
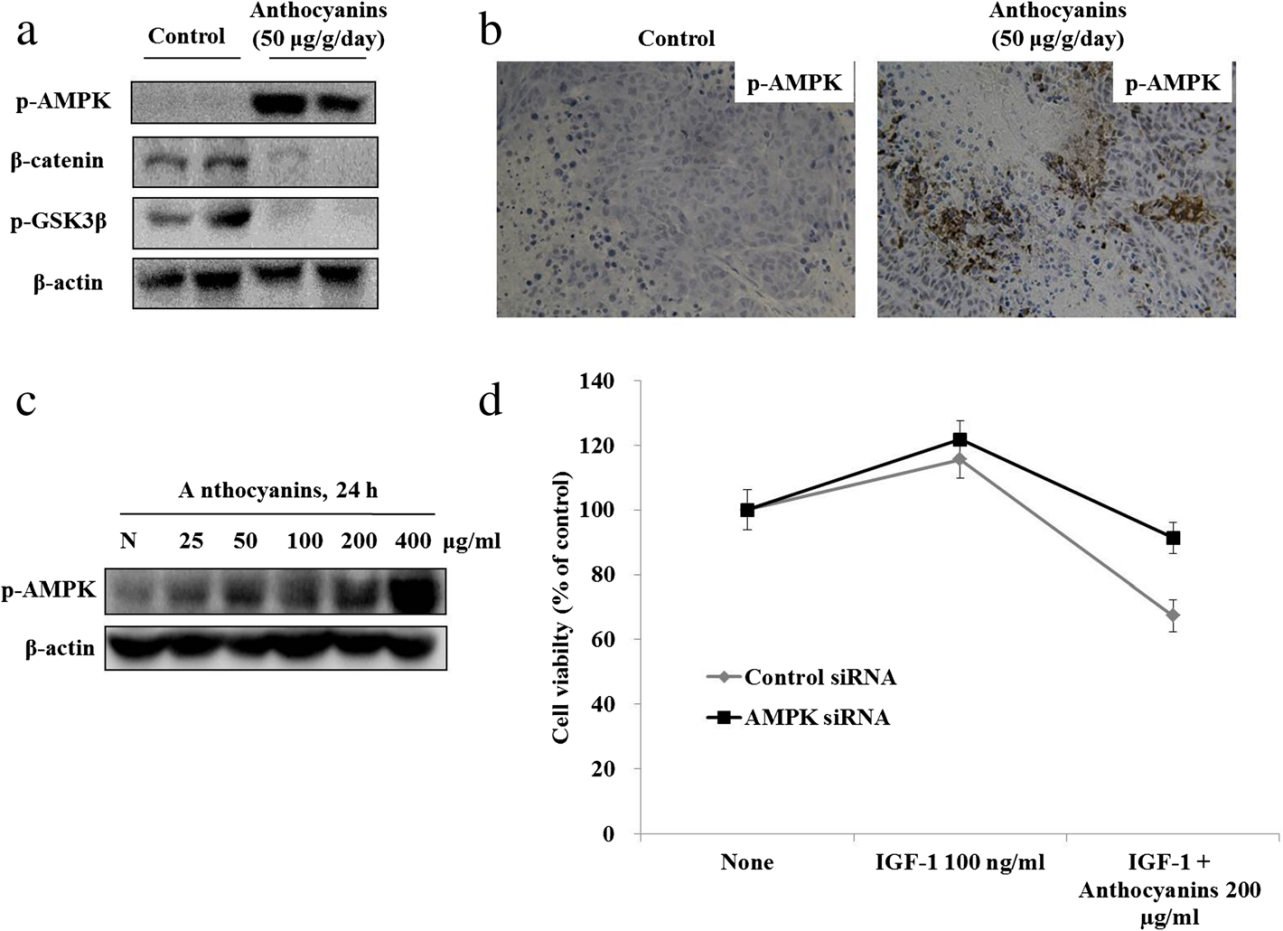
**Anthocyanins regulate GSK3β, β-catenin and AMPK *in vivo *and AMPK *in vitro*. (a)** Mice were sacrificed and tumor extracts were subjected to Western blot analysis for quantification of p-GSK3β and β-catenin levels. **(b)** The level of p-AMPK was also measured by immunohistochemical analysis. **(c)** Cells were treated with anthocyanins (25–400 μg/ml) for 24 h and total proteins were subjected to Western blot analysis using antibodies against phospho-AMPK and β-actin (loading control). **(d)** Cells were transiently transfected with AMPK siRNA or non-specific siRNA (control) for 72 h, treated with anthocyanins for 48 h, and assessed for viability using the MTT assay. Cell viability was calculated as a percentage of viable control siRNA-transfected cells.

We analyzed the molecular changes between the control and anthocyanin-treated groups. The phosphorylation of GSK3β and increased expression of β-catenin were significantly suppressed in the anthocyanin-treated group relative to that in the control group (Figure 2a). Immunohistochemical analysis showed that treatment with anthocyanins increased the level of p-AMPK compared to the controls (Figure 2b). *In vitro*, anthocyanins activated AMPK (Figure 2c), whereas siRNA-mediated knockdown of AMPK activity suppressed cancer cell growth (Figure 2d).

### Anthocyanins decrease cell invasion at least in part through AMPK activation

**Figure 3:**
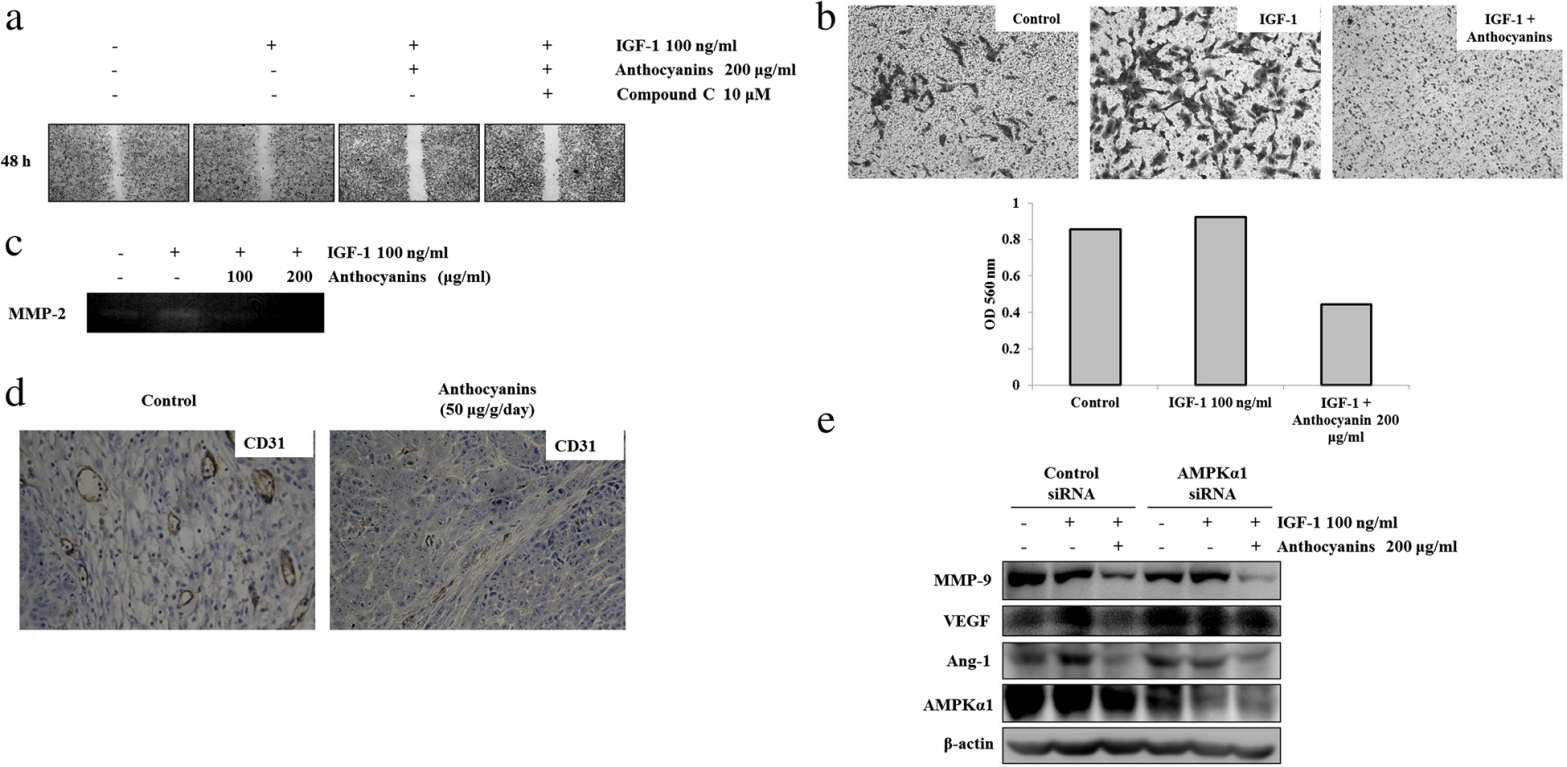
**Anthocyanins inhibit cell invasion by regulating metastasis-related proteins *in vivo *and *in vitro*. (a)** Hep3B cells were serum starved for 12 h and grown to confluence on a 6-well plate. Monolayers were wounded with a pipette tip, pretreated with IGF-1 (100 ng/ml) with or without Compound C for 30 min, and then treated with anthocyanins for 24 h. Images of wound closure were captured under a phase-contrast microscope after 24 h. **(b)** Hep3B cells were pretreated with IGF-1 (100 ng/ml) for 30 min, treated with anthocyanins for 24 h, and plated onto the apical side of Matrigel-coated filters in serum-free medium with or without IGF-1 and/or anthocyanins. Medium containing 2% FBS was placed in the basolateral chamber to act as a chemo-attractant. After 24 h, cells on the apical side were wiped off using a Q-tip, and cells that had migrated to the bottom of the filter were stained using crystal violet, and then photographed. **(c)** Cells were incubated with anthocyanins as described in **(c)** and Figure 4c, medium was collected, and MMP-2 activity was measured by zymography. **(d)** Mice were sacrificed, tumor extracts were prepared, and immunohistochemical analysis was used to quantify CD31 expression **(e)** Hep3B cells were transiently transfected with AMPK siRNA or non-specific siRNA for 72 h, pretreated with IGF-1 (100 ng/ml) for 30 min, treated with anthocyanins (200 μg/ml) for 24 h, and then subjected to Western blot analysis using antibodies against MMP-9, VEGF, Ang-1, AMPK and β-actin (loading control).

Tumor invasion is regulated by a multi-step biological process that involves cell motility, tube-like structure formation, matrix degradation, and cell migration. Previous reports have revealed that IGF-1 triggers cell migration and invasion in human endothelial cells and animal models [19]. Thus, we examined the effects of anthocyanins on invasion in IGF-1-treated Hep3B cells. We first performed a wound-healing experiment *in vitro* and found that IGF-1-induced cancer cell motility (as assessed by wound healing) was suppressed by treatment with 200 μg/ml anthocyanin (Figure 3a). To examine a possible correlation between anthocyanin-induced AMPK inhibition and wound healing effect, Hep3B cells were treated with the AMPK inhibitor Compound C. Compound C increased wound healing in anthocyanin-treated Hep3B cells (Figure 3b). Using a Boyden chamber invasion assay, we next examined whether anthocyanins decreased the invasiveness of IGF-1-treated Hep3B cells. As shown in Figure 3b, IGF-1 treatment increased cell invasiveness, but this effect was reduced by co-treatment with anthocyanins. During cell migration, which is an initial step of invasion, the gelatinase activities of MMP-2 and MMP-9 largely destroy the basal membrane of the cell. We tested the effects of anthocyanins on gelatinase activities of MMP-2 to exclude the possibility that IGF-1 increased MMP-2 expression; however, anthocyanins decreased IGF-1-stimulated MMP-2 activity (Figure 3c). These results suggest that anthocyanins have potential anti-migrative activity against hepato-carcinoma cells.

Cluster of differentiation 31 (CD31; also known as platelet endothelial cell adhesion molecule 1 or PECAM-1) is expressed in certain tumors, including epithelioid sarcoma-like hemangioendothelioma, other vascular tumors, histiocytic malignancies, and plasmacytomas. CD31 can help to evaluate the degree of tumor angiogenesis, which can indicate a rapidly growing tumor. Because malignant endothelial cells also commonly retain the antigen, CD31 immunohistochemistry can also be used to demonstrate both angiomas and angiosarcomas [20]. We observed reduced CD31 expression in anthocyanin-treated hepato-carcinoma tumors compared to control tumors *in vivo* (Figure 3d). Anthocyanins did not decrease the level of VEGF; however, MMP-9 and Ang-1 decreased in the absence of AMPK (Figure 3e).

### Anthocyanin-induced activation of AMPK inhibits GSK3β/β-catenin-mediated signaling in IGF-1-stimulated Hep3B cells

**Figure 4:**
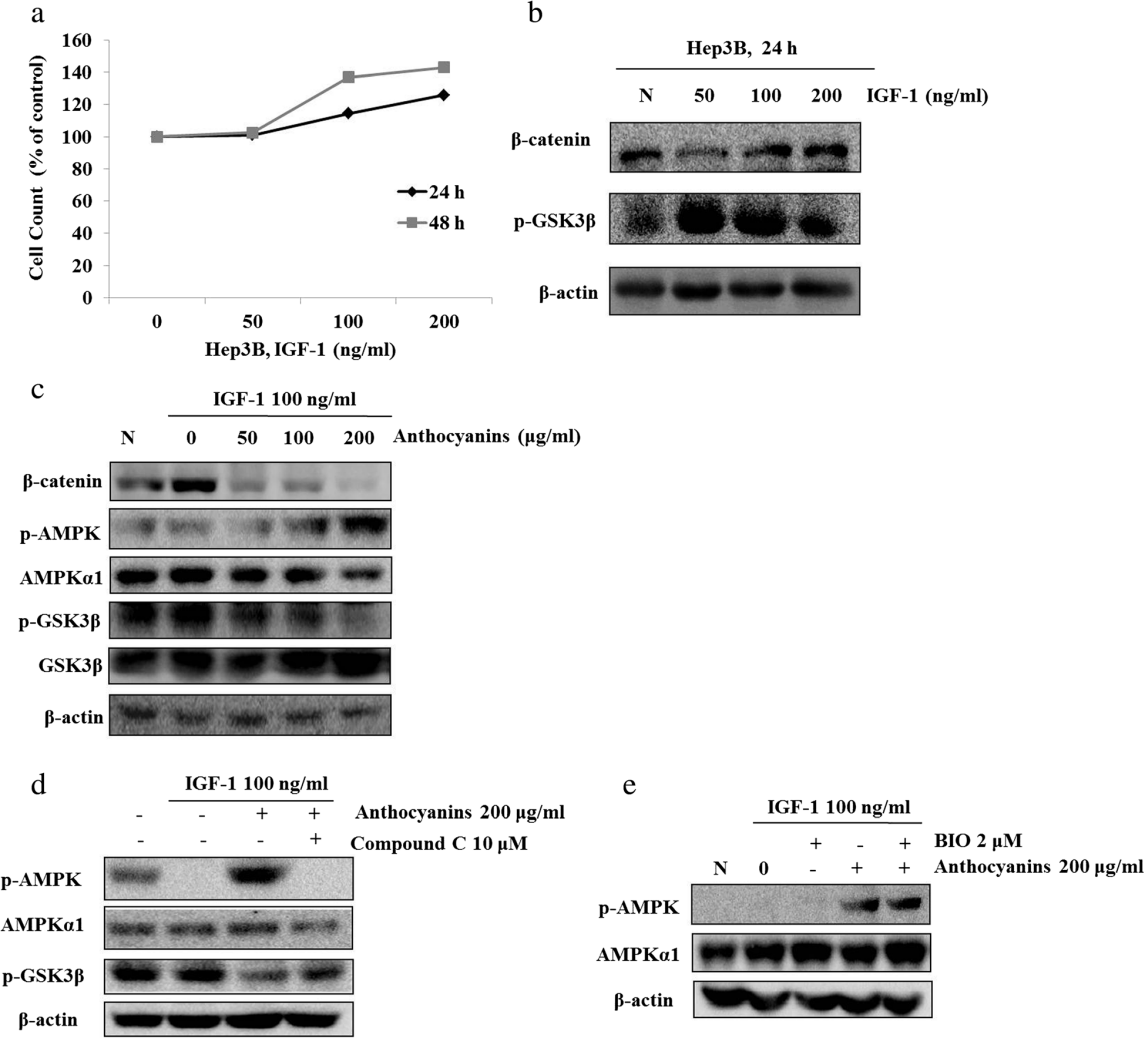
**Anthocyanins inhibit GSK3β and β-catenin in an AMPK-dependent manner. (a)** Hep3B cells were serum-starved for 12 h, treated with IGF-1 (50–200 ng/ml) for 24 or 48 h, and then stained with trypan blue. Viable cells were counted under a hemacytometer. **(b)** Hep3B cells were serum-starved for 12 h, treated with IGF-1 (50–200 ng/ml) for 24 h, and then subjected to Western blot analysis using antibodies against p-GSK3β, β-catenin and β-actin (loading control). **(c)** Hep3B cells were serum-starved for 12 h, pretreated with IGF-1 (100 ng/ml) for 30 min, and treated with anthocyanins (50–100 μM) for 24 h. Total proteins were subjected to Western blot analysis using antibodies against p-GSK3β, GSK3β, p-AMPK, AMPK, β-catenin and β-actin (loading control). **(d)** Hep3B cells were serum-starved (for 12 h), pretreated with IGF-1 (100 ng/ml) and 10 μM of Compound C for 30 min, and then treated with anthocyanins for 24 h. Western blot analysis was used to compare protein levels. **(e)** Hep3B cells were serum-starved for 12 h, pretreated with IGF-1 (100 ng/ml) and 2 μM of BIO for 30 min, and treated with anthocyanins for 24 h. Western blot analysis was used to compare protein levels.

Several reports have suggested that IGF-1 increases β-catenin transcription activity through inhibition of the destroyed Axin/APC/GSK3β complex and increases cell proliferation [21]. To elucidate the mechanism of AMPK-mediated inhibition of cell growth in anthocyanin-treated cells, we next examined the effects of IGF-1-induced Hep3B cell growth on the activation of GSK3β/β-catenin signaling. IGF-1-stimulated cell growth was associated with enhanced phosphorylation of GSK, an inhibitor, and increased expression of β-catenin (Figure 4a and b). We next analyzed these effects in anthocyanin-treated cells and found that anthocyanins at concentrations of >100 μg/ml effectively inhibited the IGF-1-stimulated increase in GSK3β phosphorylation and β-catenin expression and enhanced the phosphorylation of AMPK in these cells (Figure 4c). To examine the regulatory mechanism between AMPK and GSK3β, Hep3B cells were treated with specific inhibitors of AMPK or GSK3β (Compound C or BIO, respectively) and anthocyanins. AMPK increased the anthocyanin-induced inhibition of GSK3β activity compared to anthocyanin treatment alone (Figure 4d), whereas BIO decreased the anthocyanin-induced inhibition of GSK3β activity but did not affect the activation of AMPK (Figure 4e). These results suggest that GSK3β has an inhibitory effect on AMPK in Hep3B cells and that the anthocyanin-induced suppression of GSK3β activity can increase AMPK activity.

## Discussion

We detected a significant reduction in the phosphorylation of GSK3β in anthocyanin-treated hepato-carcinoma cells, which might result in diminished β-catenin expression. The dephosphorylation of GSK3β may account for the destruction of β-catenin, as well as the attenuated transcription of oncogenes [22]. GSK3β is a significant cancer control molecule; it controls the degradation of β-catenin by phosphorylating serine 33/37 of cytosolic β-catenin, and it may also be involved in crosstalk with the APC/CK1/Axin complex, which is important in cancer cell proliferation and metastasis^4–7^. Inactivation (i.e., phosphorylation) of GSK3β can inhibit the nuclear translocation of β-catenin, decreasing the transcription responsible for cell proliferation [23]. Increased expression of β-catenin has been observed in several cancer types, including hepato-carcinoma [24].

A large body of evidence suggests that AMPK activation is associated with the inhibition of cancer cell proliferation and metastasis [25]. Here, we report that anthocyanins activate AMPK expression *in vivo* and *in vitro*, dramatically inhibit *in vitro* hepato-carcinoma cell invasion, and down-regulate metastasis-related signaling molecules such as VEGF, MMP-9, and Ang-1. We also found that anthocyanins inhibited cell migration and invasion by inhibiting MMP-2 *in vitro* and CD31 (a panendothelial marker) *in vivo*. Several signaling pathways downstream of AMPK have been shown to control cancer cell proliferation and metastasis, including the AMPK/MAPK, JNK/STAT3, and AMPK/p53 pathways [25-28].

Here, we used AMPK and GSK3β inhibitors to investigate whether the ability of AMPK to regulate the GSK3β/β-catenin pathway is required for anthocyanins to exert their anti-proliferative and anti-metastatic functions. When AMPK was inhibited by Compound C, anthocyanin treatment failed to decrease p-GSK3β, indicating that AMPK activation is required for anthocyanins to regulate p-GSK3β. In contrast, BIO-mediated inhibition of GSK3β stimulated β-catenin but did not significantly alter AMPK activation. Taken together, our findings suggest that AMPK-mediated regulation of GSK3β is responsible for the anthocyanin-induced decrease in the proliferation and metastatic potential of hepato-carcinoma cells.

## Conclusion

This study provides evidence that the interplay between GSK3β and β-catenin, regulated by AMPK signaling, may contribute to cancer cell proliferation and metastasis. Given that the down-regulation of GSK3β/β-catenin activity appears to decrease the tumorigenic potential of hepato-carcinoma cells, molecules that are involved in the AMPK-mediated regulation of GSK3β, such as anthocyanins, could serve as potential therapeutic targets for the suppression of cancer progression and metastasis.

## Competing interests

The authors declare that there are no competing interests.

## Authors’ contributions

SY, YK, and WS carried out the experiments. SY, YK, OJ, and YM designed and conceived the study. SY and OJ wrote the paper. All authors read and approved the final manuscript.

## Pre-publication history

The pre-publication history for this paper can be accessed here:

http://www.biomedcentral.com/1472-6882/14/109/prepub

## References

[B1] Llovet JM Bruix J Molecular targeted therapies in hepatocellular carcinoma Hepatology 2008 48 1312 1327 18821591 10.1002/hep.22506 PMC2597642

[B2] Yasuhara R Yuasa T Williams JA Byers SW Shah S Pacifici M Iwamoto M Enomoto-Iwamoto M Wnt/beta-catenin and retinoic acid receptor signaling pathways interact to regulate chondrocyte function and matrix turnover J Biol Chem 2010 285 317 327 19858186 10.1074/jbc.M109.053926 PMC2804179

[B3] Friedl P Wolf K Tumour-cell invasion and migration: diversity and escape mechanisms Nat Rev Cancer 2003 3 362 374 12724734 10.1038/nrc1075

[B4] Polakis P Wnt signaling and cancer Genes Dev 2000 14 1837 1851 10921899

[B5] Rubinfeld B Albert I Porfiri E Fiol C Munemitsu S Polakis P Binding of GSK3β to the APC-β-catenin complex and regulation of complex assembly Science 1996 272 1023 1026 8638126 10.1126/science.272.5264.1023

[B6] Grimes CA Jope RS The multifaceted roles of glycogen synthase kinase 3β in cellular signaling Prog Neurobiol 2001 65 391 426 11527574 10.1016/s0301-0082(01)00011-9

[B7] Yoshida R Kimura N Harada Y Ohuchi N The loss of E-cadherin, alpha- and beta-catenin expression is associated with metastasis and poor prognosis in invasive breast cancer Int J Oncol 2001 18 513 520 11179480

[B8] Surh YJ Cancer chemoprevention with dietary phytochemicals Nat Rev Cancer 2003 3 768 780 14570043 10.1038/nrc1189

[B9] Kong JM Chia LS Goh NK Chia TF Brouillard R Analysis and biological activities of anthocyanins Phytochemistry 2003 64 923 933 14561507 10.1016/s0031-9422(03)00438-2

[B10] Zafra-Stone S Yasmin T Bagchi M Chatterjee A Vinson JA Bagchi D Berry anthocyanins as novel antioxidants in human health and disease prevention Mol Nutr Food Res 2007 51 675 683 17533652 10.1002/mnfr.200700002

[B11] Devi PS Kumar MS Das SM DNA Damage Protecting Activity and Free Radical Scavenging Activity of Anthocyanins from Red Sorghum (Sorghum bicolor) Bran Biotechnol Res Int 2012 doi:10.1155/2012/258787 10.1155/2012/258787 PMC3286891 22400119

[B12] Shin DY Ryu CH Lee WS Kim DC Kim SH Hah YS Lee SJ Shin SC Kang HS Choi YH Induction of apoptosis and inhibition of invasion in human hepatoma cells by anthocyanins from meoru Ann N Y Acad Sci 2009 1171 137 148 19723048 10.1111/j.1749-6632.2009.04689.x

[B13] Shin DY Lee WS Kim SH Kim MJ Yun JW Lu JN Lee SJ Tsoy I Kim HJ Ryu CH Kim GY Kang HS Shin SC Choi YH Anti-invasive activity of anthocyanins isolated from Vitis coignetiae in human hepatocarcinoma cells J Med Food 2009 12 967 972 19857058 10.1089/jmf.2008.1338

[B14] Shih PH Yeh CT Yen GC Effects of anthocyanidin on the inhibition of proliferation and induction of apoptosis in human gastric adenocarcinoma cells Food Chem Toxicol 2005 43 1557 1566 15964118 10.1016/j.fct.2005.05.001

[B15] Kim SM Chung MJ Ha TJ Choi HN Jang SJ Kim SO Chun MH Do SI Choo YK Park YI Neuroprotective effects of black soybean anthocyanins via inactivation of ASK1-JNK/p38 pathways and mobilization of cellular sialic acids Life Sci 2012 90 874 882 22575822 10.1016/j.lfs.2012.04.025

[B16] Xia M Ling W Zhu H Ma J Wang Q Hou M Tang Z Guo H Liu C Ye Q Anthocyanin attenuates CD40-mediated endothelial cell activation and apoptosis by inhibiting CD40-induced MAPK activation Atherosclerosis 2009 202 41 47 18495129 10.1016/j.atherosclerosis.2008.04.005

[B17] Huang C Zhang D Li J Tong Q Stoner GD Differential inhibition of UV-induced activation of NF kappa B and AP-1 by extracts from black raspberries, strawberries, and blueberries Nutr Cancer 2007 58 205 212 17640167 10.1080/01635580701328453

[B18] Yun JW Lee WS Kim MJ Lu JN Kang MH Kim HG Kim DC Choi EJ Choi JY Kim HG Lee YK Ryu CH Kim G Choi YH Park OJ Shin SC Characterization of a profile of the anthocyanins isolated from Vitis coignetiae Pulliat and their anti-invasive activity on HT-29 human colon cancer cells Food Chem Toxicol 2010 48 903 909 20060025 10.1016/j.fct.2009.12.031

[B19] Coombes BK Mahony JB Chlamydia pneumoniae infection of human endothelial cells induces proliferation of smooth muscle cells via an endothelial cell-derived soluble factor(s) Infect Immun 1999 67 2909 2915 10338498 10.1128/iai.67.6.2909-2915.1999 PMC96599

[B20] Newman PJ Berndt MC Gorski J White GC 2nd Lyman S Paddock C Muller WA PECAM-1 (CD31) cloning and relation to adhesion molecules of the immunoglobulin gene superfamily Science 1990 247 1219 1222 1690453 10.1126/science.1690453

[B21] Desbois-Mouthon C Cadoret A Blivet-Van Eggelpoël MJ Bertrand F Cherqui G Perret C Capeau J Insulin and IGF-1 stimulate the beta-catenin pathway through two signalling cascades involving GSK-3beta inhibition and Ras activation Oncogene 2001 20 252 259 11313952 10.1038/sj.onc.1204064

[B22] Park S Choi J Inhibition of beta-catenin/Tcf signaling by flavonoids J Cell Biochem 2010 110 1376 1385 20564233 10.1002/jcb.22654

[B23] Hou X Arvisais EW Davis JS Luteinizing hormone stimulates mammalian target of rapamycin signaling in bovine luteal cells via pathways independent of AKT and mitogen-activated protein kinase: modulation of glycogen synthase kinase 3 and AMP-activated protein kinase Endocrinology 2010 151 2846 2857 20351317 10.1210/en.2009-1032 PMC2875818

[B24] Fujito T Sasaki Y Iwao K Miyoshi Y Yamada T Ohigashi H Ishikawa O Imaoka S Prognostic significance of beta-catenin nuclear expression in hepatocellular carcinoma Hepatogastroenterology 2004 51 921 924 15239213

[B25] Kim HS Kim MJ Kim EJ Yang Y Lee MS Lim JS Berberine-induced AMPK activation inhibits the metastatic potential of melanoma cells via reduction of ERK activity and COX-2 protein expression Biochem Pharmacol 2012 83 385 394 22120676 10.1016/j.bcp.2011.11.008

[B26] Wu X Yan Q Zhang Z Du G Wan X Acrp30 inhibits leptin-induced metastasis by downregulating the JAK/STAT3 pathway via AMPK activation in aggressive SPEC-2 endometrial cancer cells Oncol Rep 2012 27 1488 1496 22327423 10.3892/or.2012.1670

[B27] Koide N Nishio A Hiraguri M Hanazaki K Adachi W Amano J Coexpression of vascular endothelial growth factor and p53 protein in squamous cell carcinoma of the esophagus Am J Gastroenterol 2001 96 1733 1740 11419822 10.1111/j.1572-0241.2001.03866.x

[B28] Liang KW Yin SC Ting CT Lin SJ Hsueh CM Chen CY Hsu SL Berberine inhibits platelet-derived growth factor-induced growth and migration partly through an AMPK-dependent pathway in vascular smooth muscle cells Eur J Pharmacol 2008 590 343 354 18590725 10.1016/j.ejphar.2008.06.034

